# Common occurrence of *Cryptosporidium hominis* in asymptomatic and symptomatic calves in France

**DOI:** 10.1371/journal.pntd.0006355

**Published:** 2018-03-29

**Authors:** Romy Razakandrainibe, El Hadji Ibrahima Diawara, Damien Costa, Laetitia Le Goff, Denis Lemeteil, Jean Jacques Ballet, Gilles Gargala, Loïc Favennec

**Affiliations:** Normandie Université, UNIROUEN, EA3800, CNR laboratoire expert Cryptosporidiose, Rouen, France; University of Iowa, UNITED STATES

## Abstract

**Background:**

*Cryptosporidium* spp. infections are the most frequent parasitic cause of diarrhea in humans and cattle. However, asymptomatic cases are less often documented than symptomatic cases or cases with experimentally infected animals. *Cryptosporidium (C*.*) hominis* infection accounts for the majority of pediatric cases in several countries, while *C*. *parvum* is a major cause of diarrhea in neonatal calves. In cattle *Cryptosporidium* spp. infection can be caused by *C*. *parvum*, *C*. *bovis*, *C*.*andersoni* and *C*. *ryanae*, and recently, reports of cattle cases of *C*. *hominis* cryptosporidiosis cases suggest that the presence of *C*. *hominis* in calves was previously underestimated.

**Methodology/Principal findings:**

From February to November 2015, *Cryptosporidium* spp. infected calves were detected in 29/44 randomly included farms from 5 geographic regions of France. *C*. *hominis* and *C*. *parvum* were found in 12/44 and 26/44 farms, respectively with higher *C*. *hominis* prevalence in the western region. In 9 farms, both *C*. *parvum* and *C*. *hominis* were detected. Eighty-six of 412 (73/342 asymptomatic and 13/70 symptomatic) one to nine-week-old calves shed *C*. *hominis* or *C*. *parvum* oocysts (15 and 71 calves, respectively), with no mixed infection detected. The predominant *C*. *hominis* IbA9G3 genotype was present in all regions, and more frequent in the western region. An incompletely characterized Ib, and the IbA13G3, IbA9G2 and IbA14G2 genotypes were present only in the western region. For *C*. *parvum*, the most frequent genotype was IIaA16G3R1 with no geographic clustering. Most *C*. *hominis* infected calves were asymptomatic, with some exceptions of IbA9G2 and IbA9G3 isolates, while *C*. *parvum* IIaA16G3R1 was associated with symptoms.

**Conclusions/Significance:**

Present results indicate for the first time that in several geographic regions of France, *C*. *hominis* was present in about one fifth of both asymptomatic and symptomatic infected calves, with isolated genotypes likely associated with human infection. Further investigations are aimed at documenting direct or indirect transmissions between livestock and humans.

## Author summary

Symptomatic infection by the Apicomplexan *Cryptosporidium* spp. is presently considered the most frequent parasitic cause of acute diarrhea in both humans (especially severe in immunocompromised individuals and infants in both developed and developing countries) and cattle (calves), while asymptomatic infections are less often documented. *Cryptosporidium (C*.*) hominis* once considered to be restricted to humans accounts for the majority of pediatric cases in several countries. *C*. *parvum* can also infect cattle as well as *C*. *bovis*, *C*. *andersoni*, and *C*. *ryanae*. Recently, cattle *C*. *hominis* cryptosporidiosis has been reported, suggesting that the presence of *C*. *hominis* in calves was previously underestimated. The aim of this work was to characterize *Cryptosporidium* spp. infection in both asymptomatic and symptomatic dairy and beef calves from Metropolitan France. From February to November 2015, *C*. *parvum* or *C*. *hominis* infected calves were detected in farms from 5 geographic regions of France. Surprisingly, C. *hominis* was present in about one fifth of *Cryptosporidium* spp. infected calves, and exhibited genotypes which were previously reported in human and nonhuman primate. Further investigations are aimed at documenting direct or indirect *C*. *hominis* transmissions between and among livestock and humans.

## Introduction

*Cryptosporidium* spp. are Apicomplexa which include parasite species causing asymptomatic to severe gastrointestinal infections in a wide range of vertebrate hosts, and exhibiting varying degrees of host adaptation [1]. Previously, information on cryptosporidial host restriction of natural cryptosporidial infection was usually obtained from animal and human cases for which clinical symptoms, ages and immune statuses were recorded. However, evidence of asymptomatic sustained or transitory infection, and the role of additional parameters such as parasite detection methods, host’s genetic background, co-infection and environmental factors such as climate, seasons and socioeconomic status were less documented [2]. For some cryptosporidial host specialisations, information is presently limited to experimentally infected immunocompetent or immunosuppressed laboratory animals [3].

In humans, cryptosporidiosis is presently identified as the most frequent zoonotic cause of parasitic diarrhea, especially severe in immunocompromised individuals and infants in both developed and developing countries [4–7]. In addition to 8 others sporadically observed species, *C*. *hominis*, once considered to be restricted to humans, and *C*. *parvum*, of which some isolate genotypes also infect ruminants, account for more than 90% of reported human cases worldwide [8–10]. There is equal or higher prevalence of *C*. *hominis* than *C*. *parvum* in humans in many parts of the world except in Europe where *C*. *parvum* largely prevails, likely reflecting the ratios of human to animal sources of anthroponotic *C*. *hominis* and anthropozoonotic *C*. *parvum* contamination, respectively [11–15].

In cattle, the main symptom of cryptosporidiosis is watery and profuse acute diarrhea which can be associated with dehydration, anorexia, and impaired growth [16]. It was previously established that cattle can be infected by at least 4 *Cryptosporidium* species, *i*.*e*. *C*. *parvum*, *C*. *bovis*, *C*. *andersoni*, and *C ryanae* [17, 18]. In France, *C*. *parvum*, *C*. *bovis* and *C*. *andersoni* predominate in newborn and older calves, respectively, *C*. *parvum* infection is identified as a major cause of diarrhea in newborn calves of less than one month old, with economically significant morbidity and mortality. However, detailed epidemiology on the occurrence of viable oocysts from normal feces of asymptomatic calves is unknown [19, 20]. Recently, a limited number of observations reported of cattle cryptosporidiosis due to *C*. *hominis* have been reported in Australasia, Asia, Africa and Europe, suggesting that the presence of *C*. *hominis* in calves was previously underestimated in studies on diarrheic and adult animals [21–27].

The aim of this work was to document the prevalence of *Cryptosporidium* spp oocysts in calves from five different geographic regions of Metropolitan France. Farms were randomly included in the study, the clinical status of each animal was recorded, and the presence of calves with *Cryptosporidium* spp. oocysts in feces was investigated. Isolates were genetically characterized for their synzootic and zoonotic potentials.

## Methods

### Calves and fecal samples

From February to November 2015, 412 calves aged from 1 to 9 weeks were selected in 44 farms from a national list of farms under regular veterinarian survey (16 veterinary offices, from 1 to 4 farm(s) per office). Farms were randomly selected, and within farms, calves aged from 1 to 9 weeks were randomly selected. Selected farms were situated in 14 “départements” (an administrative sub-region) distributed in 5 geographic regions of Metropolitan France: western (Côtes d'Armor, Ille-et-Vilaine, Morbihan), central western (Vendée, Deux Sèvres, Mayenne), northeastern (Pas-de- Calais, Moselle), southwestern (Landes, Pyrénées atlantiques, Tarn, Hautes-Pyrénées), and central (Puy-de-Dôme, Allier). For each calf, the clinical status was evaluated and recorded by all veterinarians at the time of sampling as follows: presence or absence of digestive symptoms such as diarrhea and abdominal bloating and/or respiratory symptoms, and evaluation of the general condition as follows: "Normal general condition: shiny hair coat, regular appetite; impaired general condition: delayed growth, dull hair coat, capricious appetite; Poor general condition: dull hair coat, capricious appetite, marked growth retardation."

From each farm (housing from 11 to 50 calves), feces samples were obtained by veterinarians from 5–10 calves by rectal stimulation. Most farms were dairy farms and breeds consisted of Salers, Holstein, Charolais, Montbeliard, Blonde d'Aquitaine, Parthenaise, Limousine and the Belgian Blue Breed (BBB). In all farms, calves were kept in semi-intensive farming systems and separated from their dams.

### Microscopic detection of *Cryptosporidium* spp. oocysts in calf feces

The presence of *Cryptosporidium* spp. oocysts was microscopically determined by the same experienced clinical parasitologists using Bailenger type feces concentration method [28] and Heine staining [29]. The presence of other intestinal parasites (*Giardia*, *Strongyloides*, and coccidia) detected in some of the calves using various methodologies was not considered in the present study.

### *Cryptosporidium* speciation and genotyping

All samples were subjected to molecular analysis for speciation and genotyping of speciation positive samples. Before DNA isolation, feces were subjected to a pre-treatment with a mechanical lysis in Lysing Matrix A Tubes (garnet matrix and ¼ ceramic spheres) (Qiagen, CA, USA) with the Fastprep-24 device and transferred into 2 ml Eppendorf tube prior to thermal shock lysis (6 freeze-thaw cycles). Samples were placed in an ultrasonic bath for sonication (3x20 sec bursts). In accordance with the manufacturer's instructions, a modified QIAamp Stool Mini Kit (Qiagen, CA, USA) was used to isolate DNA from the pre-treated samples. All centrifugation steps were performed at RT (20–25°C), at 14.000 rpm. Eight hundred μL/tube of ASL buffer was added, and tubes were heated at 99°C for 15 min.

For speciation, a 18S rRNA gene sequence was amplified using a nested PCR and restriction digestion of the secondary product with SspI (NEB, MA, USA) and VspI (NEB, MA, USA) was performed [30]. Briefly, for the primary PCR step, a PCR product (about 1,325 bp long) was amplified by using primers 5-TTCTAGAGCTAATACATGCG-3 and 5-CCCTAATCCTTCGAAACAGGA-3. For the secondary PCR step, by using 5 μl of the primary PCR product and primers 5-GGAAGGGTTGTATTTATTAGATAAAG-3’ and 5-AAGGAGTAAGGAACAACCTCCA-3 a PCR product (819 to 825 bp long, depending on the species) was amplified.

Each PCR mixture (total volume, 50 μl) contained 5 μl of 10X DreamTaq Buffer, each deoxynucleoside triphosphate at a concentration of 0.2mM, each primer at a concentration of 100 nM, 2.5 U of DreamTaq polymerase, and 5μL of DNA template. Then, 1.25μL of DMSO (100%) was added to the mixture.

A total of 40 cycles, each consisting of 94°C for 45 s, 55°C for 45 s, and 72°C for 1 min, were performed. An initial hot start at 94°C for 3 min and a final extension step at 72°C for 7 min were also included. Each amplification run included a negative control (PCR water) and two positive controls (genomic DNA from *C*. *parvum oocysts purchased from* Waterborne Inc., *and C*. *hominis genomic DNA* from fecal specimen collected at Rouen University Hospital). Products were visualized in 2% agarose gels using ethidium bromide staining and identification was confirmed by sequencing. Positive samples were further genotyped by DNA sequencing of the *gp60* gene amplified by a nested PCR following the protocol described by Sulaiman *et al*. [31] (2005). All Amplification experiments were repeated at least thrice to check reproducibility.

Purified PCR products were sequenced in both directions on an ABI 3500 sequencer analyzer (Applied Biosystems, CA, USA) by using the secondary PCR primers and the BigDye Terminator v3.1 Cycle Sequencing Kit (Applied Biosystems, CA, USA). The obtained sequences were inspected using the 4 peaks software (https://nucleobytes.com/4peaks/index.html), edited with the BioEdit sequence alignment editor (version 7.2.5), and analyzed for DNA database search and comparisons using the BLAST server (www.ncbi.nlm.nih.gov/BLAST). Genotypes were named using the established *gp60* genotype nomenclature [31].

### Statistical analysis

Statistical analyses were performed using the chi-square (χ2) test or Fisher’s exact test as appropriate using the Number Cruncher Statistical System (NCSS), version 2000 to determine the association between the prevalence of *Cryptosporidium* infection vs regions, and sampling periods. A *p value* <0.05 was considered statistically significant.

### Ethics statement

Before carrying out this work, informed written authorization to perform and anonymously publish the present epidemiological study was obtained from all cattle owners and veterinarians. Clinical examination of calves and stool harvest were part of routine breeding and veterinary procedures, without any invasive, traumatic or specific containment method. Such procedures are not qualified as animal experimentation involving vertebrate according to French laws, and no specific ethical clearing was required.

## Results

As shown in Table 1, infected calves were detected in 29/44 farms from all geographic regions and “départements” except Puy-de-Dôme and Mayenne, with no inter-regional difference in the ratios of the number of infected farms to the number of included farms. *C*. *hominis* and *C*. *parvum* were found in calves from 12/44 and 26/44 farms, respectively. In 9/44 farms, both *C*. *parvum* and *C*. *hominis* infected calves were found with no mixed infection in any animal. No mixed infection of *C*. *hominis* and *C*. *parvum* was noted in any animal.

**Table 1:** Geographic distribution of 15 *Cryptosporidium hominis* and 71 *Cryptosporidium parvum* infected calves from 44 farms in 5 regions.

Region	Département/ Region subtotal	Included farms		Included calves	*C*. *hominis* infected farms	*C*. *parvum* infected farms	*Cryptospordium* infected farms	*C*. *hominis* infected calves	*C*. *parvum* infected calves	*Cryptosporidium* infected calves
Central	Allier	3	31		1	3	3	1	12	13
	Puy-de-Dôme	4	40		0	0	0	0	0	0
	region subtotal	7	41		1	3	3	1	12	13
Western	Côtes d'Armor	1	10		0	1	1	0	2	2
	Ile-et-Vilaine	6	51		4	2	4	5	4	9
	Morbihan	6	58		4	4	4	6	7	13
	Region subtotal	13	119		8	7	9	11	11	22
Southwestern	Pyrénées Atlantiques	2	17		0	1	1	0	2	2
	Hautes-Pyrénées	1	10		0	1	1	0	2	2
	Landes	2	19		0	1	1	0	2	2
	Tarn	4	39		1	3	3	1	4	5
	Region subtotal	9	85		1	6	6	1	10	11
Central western	Mayenne	1	5		0	0	0	0	0	0
	Deux-Sèvres	4	35		0	3	3	0	9	9
	Vendée	3	30		1	2	2	1	5	6
	Region subtotal	8	70		1	5	5	1	14	15
Northwestern	Moselle	4	37		0	3	3	0	13	13
	Pas-de Calais	3	30		1	2	3	1	11	12
	Region subtotal	7	67		1	5	6	1	24	25
Total		44	412		12	26	29	15	71	86

Results given in numbers of farms or calves.

Eighty-six of 412 included calves exhibited *Cryptosporidium* spp. oocysts in feces, of which 15 and 71 had *C*. *hominis* and *C*. *parvum* infection respectively. There were no inter-regional differences in the ratio of the number of infected calves to the number of included calves (p = 0.839). In the western region, the ratio of *C*. *hominis* infected farms (8/13) was higher than in all other geographic regions (p = 0.018), while no interregional difference was found for *C*. *parvum* (p = 0.122). Infections were found in calves from 2 weeks to 7 weeks of age (3 to 7 weeks and 2 to 7 weeks for *C*. *hominis* and *C*. *parvum* cases, respectively).

As shown in Table 2, *C*. *hominis* IbA9G3 genotype isolates were predominant and present in all geographic regions. The incompletely characterized Ib and the IbA13G3, Ib A9G2 and IbA14G2 genotypes were only represented in the western region.

**Table 2:** Geographic distribution of *15 Cryptosporidium hominis* genotypes from infected calves in 5 regions.

Geographic region	Département	Calves with *C*. *hominis* genotype				
Region		IbA9G3	Ib	IbA13G3	IbA9G2	IbA14G2
Central	Allier	1				
	Puy-de-Dôme					
Western	Côtes d'Armor					
	Ille-et-Vilaine	2	1	1	1	
	Morbihan	2	3			1
Southwestern	Pyrenees-Atlantiques					
	Landes					
	Tarn	1				
Central western	Mayenne					
	Deux-Sèvres					
	Vendee	1				
Northwestern	Moselle					
	Pas-de-Calais	1				
Total		8	4	1	1	1

Results given in numbers of calves

For *C*. *parvum*, IIaA16G3R1 genotype was the most frequent with no geographic clustering (p = 0.574), and the limited number of isolates exhibiting other genotypes precluded further investigation on their geographic representation (Table 3).

**Table 3:** Geographic distribution of 71 *Cryptosporidium parvum* genotypes from infected calves in 5 regions.

Region	Département	IIaA15G3R1	IIaA16G1R1		IIaA16G3R1		IIaA17G2R1	IIaA18G3	IIaA17G1	IIaA17G2	IIaA17G3	IIaA14GR1		IIaA19G2R1	IIaA15G2R1
Central	Allier	1	3			5	2	1							
	Puy-de-Dôme														
	Region subtotal	1	3			5	2	1							
Western	Côtes d'Armor		1												1
	Ille-et-Vilaine					1	1								
	Morbihan		1			3	1			1					1
	Region subtotal		2			4	2			1					2
South-western	Pyrenees-Atlantiques					2									
	Hautes-Pyrenees					2									
	Tarn					4									
	Landes		1						1						
	Region subtotal		1			8			1						
Central western	Mayenne														
	Deux-Sèvres					6				1	2				
	Vendee	1						1					1	1	1
	Region subtotal	1				6		1		1	2		1	1	1
North western	Moselle					9		1		1	2				
	Pas-de-Calais					10	1								
	Region subtotal					19	1	1		1	2				
Total		2	6			42	5	3	1	3	4		1	1	3

Results given in numbers of calves.

No *C*. *hominis* infected calves exhibited diarrhea during the week before or at the time of stool sampling (Table 4). Two infected calves, one with genotype IbA9G2 and one with genotype IbA9G3 presented with general state alteration and non-diarrheal digestive symptoms. Ten calves infected with the most frequent *C*. *parvum* IIaA16G3R1 genotype presented with digestive symptoms, respiratory symptoms, or both, and 12 exhibited an impaired or poor general state. The limited number of observations, precluded from investigating further associations between symptoms and genotypes.

**Table 4:** Symptoms and general conditions of *Cryptosporidium hominis* and *Cryptosporidium parvum* infected calves.

	Isolate genotype	Infected calves	Symptoms				General condition		
			Asymptomatic	Digestive	Respiratory	Digestive and respiratory	Normal	Impaired	Poor
*C*. *hominis*	Ib	4	4				4		
	IbA13G3	1	1				1		
	IbA14G2	1	1				1		
	IbA9G2	1		1				1	
	IbA9G3	8	7	1			7	1	
Total		15	13	2			13	2	
*C*. *parvum*	IIaA15G3R1	2	2				2		
	IIaA16G1R1	7	6	1			6	1	
	IIaA16G3R1	42	34	3	4	1	38	2	2
	IIaA17G2R1	5	4	1			4	1	
	IIaA18G3	3	3				2	1	
	IIaA15G2R1	3	3				1	2	
	IIaA17G1	1	1				1		
	IIaA17G2	2	2				2		
	IIaA17G3	4	3	1			2	2	
	IIaA14G1R1	1	1					1	
	IIaA19G2R1	1	1				1		
Total		71	60	6	4	1	59	10	2

Results given in number(s) of calves. Each number corresponds to one calf except for calves with genotype IIaA16G3R1 of which 2/3 presented with both symptoms and impaired general state or poor conditions.

**Fig 1:**
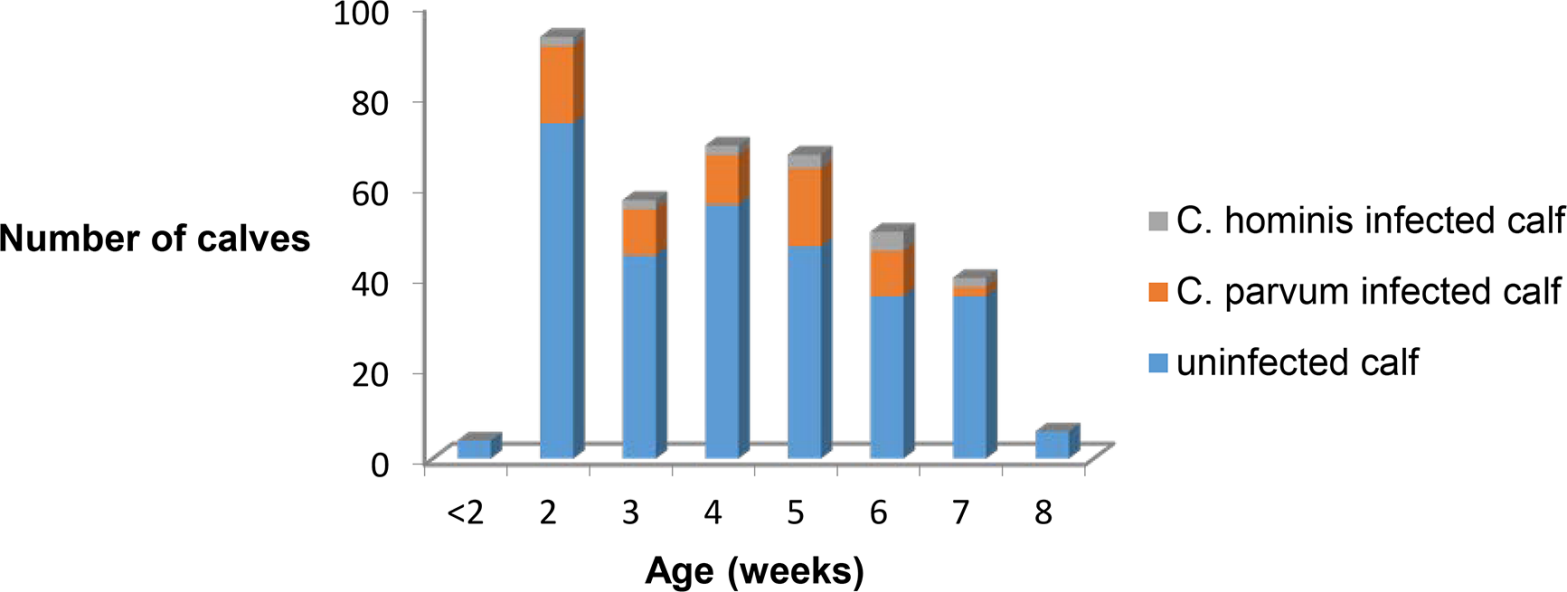
Age distribution of uninfected, *C*. *parvum* and *C*. *hominis* infected calves.

The ratios of the number of infected calves to the number of sampled calves observed during the August-September (50/216) and October-November (28/106) periods were higher than those during February to March (3/45) and April to June (5/42) periods (p = 0.010) with no difference in the respective *C*. *hominis* and *C*. *parvum* representations (the corresponding values for *C*. *hominis* were 9/50, 4/28, 2/3, and 0/5, respectively, p>0.05). No seasonal effect on *Cryptosporidium* infection prevalence, however, could be unambiguously established, taking into account that due to the yearly calving cycle, most calves were sampled during summer and autumn, and that no feces sample was obtained in January, July and December. The age distribution of *C*.*parvum* infected calves is similar to that of *C*. *hominis* infected calves (Fig 1)

## Discussion

In this work, the presence of *C*. *hominis* in calves was addressed for the first time in France by investigating both asymptomatic and symptomatic calves from randomly chosen farms in several geographic regions. Surprisingly, *C*. *hominis* was identified in about one fifth of *Cryptosporidium* spp. infected animals, a figure which to our knowledge has not been reported previously in Europe.

The random selection of farms resulted in a variety of geographic regions corresponding to different geologic and climatic characteristics with oceanic, or humid continental influences. Due to breeding procedures, most calves from all regions were sampled during summer and autumn. For each calf, age, clinical condition, farm and sampling date were recorded by veterinarians. The study was focused on calves, and unbiased sampling was confirmed by similar *C*. *hominis* infection rates of calves aged between 2 and 7 weeks. Most animals were clinically asymptomatic, none of them presenting diarrhea in the week before, or at the time of sampling, and a exhibiting digestive or respiratory symptoms and/or impaired or poor general condition consistent with clinical cryptosporidiosis. Sampling and preservation of samples were adapted to oocyst detection in calf feces: Heine-stained oocysts were detected microscopically by experienced parasitologists, and oocyst species and genotypes were determined using previously validated DNA amplification methods. *C*. *hominis* was unexpectedly detected in 15 calves. Four out of 15 *C*. *hominis* positive samples could not be identified at the genotype level, with unreadable superimposition of electrophoregrams which might result from amplification and sequencing of different genetic fragments from several genotypes present in the same sample. For both *C*. *hominis* and *C*. *parvum*, several subtypes were not seen in the same sample (32).

Farms from all “départements” except 2 presented both *C*. *hominis* and C. *parvum*-infected animals. *C*. *hominis* was present in about half of the “départements” with *Cryptosporidium* spp. infected calves, and *C*. *parvum* was present in all of them. Both the ratios of *C*. *hominis* infected farms and animals were higher in the western geographic region compared with other regions. In 9/44 farms, both *C*. *parvum* and *C*. *hominis* were detected in different calves. Although no data concerning the origin of water given to calves was available, a waterborne transmission of *C*. *hominis* is possible as illustrated by the recent occurrence of a *C*. *hominis* waterborne outbreak in France. All samples were obtained during the calving period; thus, no seasonal variation of *Cryptosporidium* species nor genotypes could be ascertained. The present unexpected high proportion of *C*. *hominis* infected calves compares with previously reported figures in New-Zealand Australia, Africa, China and Europe [21, 24, 25, 32, 33]. Several studies have established that cattle can be infected with at least 4 species (*C*. *parvum*, *C*. *bovis*, *C*. *andersoni*, and *C*. *ryanae*). *C*. *hominis* and *C*. *parvum* were the only species detected in the present study, likely due to the age of the calves, and possibly to the masking of concurrent infections by *C*. *bovis*, *C*. *ryanae or C*. *andersoni* by the predominant shedding of oocysts of other species, or to preferential PCR amplification of predominant species [17, 18, 35].

It was once generally accepted that *C hominis* primarily infects humans while *C*. *parvum* infects both human and non-human hosts, and reports of cattle *C*. *hominis* infections are few. Early observations based on *18S rDNA* gene analysis revealed mixed cases with *C*. *hominis* in heifers and cows from 2001 to 2003, and *C*. *hominis* cases in domestic cattle and goats [22, 26]. More recently, limited numbers of *C*. *hominis* isolates from cattle have been genotyped in Africa, China, New Zealand, Australia, the UK and Italy [21, 23–25, 34, 36, 37]. Besides cattle, *C*. *hominis* has been reported in sheep and goats in the UK and in foals in Brazil [38, 39].

In addition to species identification, *gp60* genotyping was performed to provide clues to symptomatic, epidemiologic, and zoonotic characteristics of isolates. Genotypes included IbA13G3, IbA9G2, and IbA9G3 and the newly described IbA14G2, and for 4 isolates, only attribution to the Ib genotype family was obtained, due to unreadable superimposition of electrophoregrams. No association between genotypes and symptoms could be determined. While 1bA9G3 was only detected in one human patient from the western region, no evidence for *C*. *hominis* case clustering and no seasonal variation could be established using genotypes. To our knowledge, none of the above *gp60* genotype sequences (IbA13G3, IbA9G2, and IbA9G3) has been previously reported in cattle. *C*. *hominis gp60* IbA10G2 genotype has been detected in Australia and New Zealand [21, 34]. The present genotyping data suggest the existence of potentially zoonotic *C*. *hominis* isolates in calves, since the 3 genotypes mentioned above have been previously described in human and non-human primates. Genotype IbA13G3 was reported in wastewaters from a densely populated urban region (Shanghai, China) [40], genotype IbA9G2 was reported in humans and genotype IbA9G3 was found in non-human primates in Kenya and China [41–43]. Such data aim at investigating associations between genotypes found in cattle and humans from the same geographic region, as reported in India for *C*. *hominis* genotype IdA15G1 found in one calf, and children from the same geographic region [44].

### Conclusions

Present results indicate for the first time that in several geographic regions of France, *C*. *hominis* was present in about one fifth of both asymptomatic and symptomatic calves, and exhibited genotypes likely linked to human infection. Cattle have been considered to be a primary reservoir for *Cryptosporidium* spp. and to play a role in transmitting zoonotic *C*. *parvum* organisms to humans [44, 45]. Results of the present study suggest that calves in France also frequently harbor *C*. *hominis* isolates which might be cause of human infections. Further investigations are aimed at determining whether the source of cattle infections was other livestock or humans, and whether the transmission was direct or indirect.
